# Muscle synergy analysis during badminton forehand overhead smash: integrating electromyography and musculoskeletal modeling

**DOI:** 10.3389/fspor.2025.1596670

**Published:** 2025-06-03

**Authors:** Raheleh Tajik, Wissem Dhahbi, Hamed Fadaei, Raghad Mimar

**Affiliations:** ^1^ Department of Biomechanics and Sports Injuries, Faculty of Physical Education and Sports Sciences, Kharazmi University, Tehran, Iran; ^2^ Research Unit “Sport Sciences, Health and Movement”, Higher Institute of Sports and Physical Education of Kef, University of Jendouba, Kef, Tunisia; ^3^ Training Department, Police College, Police Academy, Doha, Qatar

**Keywords:** biomechanical phenomena, elite athletes, joint instability, motor control, movement disorders, neuromuscular coordination, rotator cuff

## Abstract

**Introduction:**

This study aimed to quantify shoulder muscle synergies during badminton forehand overhead smash (BFOS) via non-negative matrix factorization (NMF), validate musculoskeletal (MSK) models for high-speed movements by comparing electromyography (EMG)-derived synergies with simulation results, and explore the potential of NMF-based MSK models in advancing sports science.

**Methods:**

Twenty elite badminton players (age: 24 ± 4 years; experience: 15 ± 4 years) performed maximal-effort BFOS while EMG signals from fifteen shoulder muscles were recorded. Three-dimensional motion analysis with a ten-camera Vicon system captured kinematic data at 100 Hz. A validated OpenSim upper extremity model was implemented to simulate muscle activations via static optimization. NMF extracted synergy vectors and activation coefficients from both experimental EMG and MSK modeling data.

**Results:**

Three muscle synergies accounted for >90% variance in both analyses with no significant differences in global VAF (*p* = 0.12). The first synergy (trapezius-dominant) showed 95% EMG and 97% MSK variance; the second synergy (pectoralis/anterior deltoid) exhibited 97% EMG and 94% MSK variance; the third synergy (posterior muscles) demonstrated 95% EMG and 98% MSK variance. Strong agreement between approaches was observed for both weight vectors (W1:0.81 ± 0.04, W2:0.87 ± 0.01, W3:0.88 ± 0.03) and activation coefficients (C1:0.95 ± 0.02, C2:0.98 ± 0.01, C3:0.98 ± 0.01), with differences primarily in lower trapezius activation (similarity: 0.77 ± 0.05), likely due to challenges in recording deep muscle activity through surface electromyography. These findings validate the combined experimental-computational approach for analyzing complex, high-velocity movements.

**Conclusion:**

The strong correspondence between experimental and computational synergies validates MSK modeling for analyzing neuromuscular control during high-velocity overhead movements. The identified synergies provide a framework for understanding muscle coordination during BFOS, with potential applications in targeted training program optimization and injury prevention strategies in overhead sports.

## 1 Introduction

The badminton forehand overhead smash (BFOS) represents one of the most explosive strokes in racquet sports, with elite players generating shuttlecock velocities exceeding 118 m/s (1). This remarkable velocity requires exceptional neuromuscular coordination through a kinetic chain from lower extremity to racquet-shuttlecock impact (2). The repetitive and explosive nature of the BFOS places significant stress on the shoulder complex, contributing to 1%–5% of sports-related shoulder injuries attributed to badminton (2, 3).

**Table 1:** Characteristics of the hill-type tendon-muscle model for the MSK model.

Muscle	Optimal fiber length (cm)	Peak force (N)	Tendon slack length (cm)	Pennation angle (^o^)
TRPL1	0.1127	1043	0.027	0
TRPL2	0.0832	470.4	0.032	0
TRPL3	0.1264	414.4	0.035	0
TRPL4	0.1116	201.6	0.027	0
DELT1	9.8	1218.9	9.3	22
DELT2	10.8	1103.5	11	15
DELT3	13.7	201.6	3.8	18
SUPRA	6.8	499.2	4	7
INFRA	7.6	1075.8	3.1	19
SUBSCAP	8.7	1306.9	3.3	20
TMIN	7.4	269.5	7.1	24
TMAJ	16.2	144	2	16
PECM1	14.4	444.3	0.3	17
PECM2	13.8	658.3	8.9	26
PECM3	13.8	498.1	13.2	25

DELT1 = anterior deltoid; DELT2 = medial deltoid; DELT3 = posterior deltoid; INFSP = infraspinatus; PECM1 = pectoralis major clavicular; PECM2 = pectoralis major medial; PECM3= pectoralis major inferior; LAT2 = latissimus dorsi medial; TRIlat = triceps lateral head; TRImed = triceps medial head; BIClong = biceps long head; TRP1 = upper trapezius; TRP3 = middle trapezius; TRP4 = lower trapezius; SRA = serratus anterior.

The shoulder joint complex, with its multiple articulations and extensive musculature, transfers energy from trunk to upper extremity during overhead movements while maintaining dynamic control despite limited inherent stability (4, 5). Previous investigations have documented substantial biomechanical demands during the BFOS, with elite players generating shoulder internal rotation moments of 0.85 ± 0.12 Nm/kg and peak shoulder internal rotation velocities reaching 7148°/s (6). These velocities are comparable to baseball pitching (7200°/s) (7) and exceed tennis serving (2900°/s) (7) and volleyball spiking (2594°/s) (5, 7), highlighting the sophisticated coordination strategies required (8).

The central nervous system (CNS) faces considerable computational challenges in controlling the redundant musculoskeletal system during complex movements (9). The muscle synergy hypothesis provides a theoretical framework suggesting that the CNS simplifies this control problem by activating functional groups of muscles (synergies) rather than individual muscles independently (10). This modular organization potentially reduces the dimensionality of motor control and facilitates efficient movement execution (11). Non-negative matrix factorization (NMF) has emerged as a robust computational technique for extracting underlying muscle synergies from electromyographic (EMG) data (12). This algorithm decomposes multi-muscle activation patterns into a small set of time-invariant muscle weightings (synergy vectors) and their corresponding time-varying activation coefficients (12, 13). However, recording EMG data from all relevant muscles during high-speed actions presents significant technical challenges (7, 14), particularly for deep or inaccessible muscles that contribute substantially to shoulder function during overhead movements. Within the context of overhead sports movements, previous investigations have identified consistent synergy structures across various upper extremity tasks, including reaching (7, 15), grasping (8), and throwing (8, 15). Pale et al. (16) demonstrated that approximately three to four synergies could account for over 90% of the variance in muscle activation patterns during hand grasps, with synergy structures showing strong within-subject consistency but notable inter-subject variability. Nevertheless, the application of muscle synergy analysis to high-velocity overhead sports movements remains underexplored, particularly in badminton (17).

Recent advances in musculoskeletal (MSK) modeling have enabled comprehensive analysis of complex movements through integration of anatomical, physiological, and biomechanical principles (18). These computational approaches complement experimental measurements by providing estimates of variables that cannot be directly measured *in vivo*, such as individual muscle forces, joint contact forces, and moment arms (19). The OpenSim modeling framework has gained widespread adoption for biomechanical investigation, offering validated models of the upper extremity with physiologically accurate muscle parameters (20). MSK models can generate muscle activation predictions through optimization algorithms that distribute joint moments across available muscles (21). Static optimization, for example, minimizes the sum of squared muscle activations subject to moment equilibrium constraints, providing computationally efficient estimates of muscle coordination patterns (22). However, the biological validity of these computational predictions requires thorough experimental validation, particularly for complex multi-joint movements involving rapid accelerations (23). Recent studies have demonstrated that MSK modeling can effectively capture muscle synergies during dynamic movements (18). These models have successfully replicated walking and running patterns by optimizing a small number of motor control parameters (18). However, the unique demands of the BFOS, particularly on the shoulder complex, require more targeted investigations. Several investigators have compared experimentally measured EMG signals with model-predicted activations during various tasks, reporting moderate to strong correlations in controlled movements (24). Nevertheless, the accuracy of MSK model predictions during high-velocity overhead sports movements remains incompletely characterized (25), particularly regarding muscle synergy structures.

Despite extensive research on overhead throwing biomechanics, several knowledge gaps persist. First, while individual muscle activations have been characterized during badminton smashes (25, 26), the underlying synergistic control structures remain poorly understood. Second, although MSK models have been validated for various upper extremity movements (14), their applicability to high-velocity overhead sports movements requires further investigation. Third, the relationship between experimentally measured and computationally predicted muscle synergies during complex sports movements has not been thoroughly examined (17).

The objectives of this study are threefold. First, we aim to quantify shoulder muscle synergies during the forehead overhead smash via NMF. Second, we seek to validate MSK models for high-speed movements by comparing EMG-derived synergies with simulation results. Third, we aim to explore the potential of NMF-based MSK models in advancing sports science. We hypothesized that (1) three to four muscle synergies would account for >90% of the variance in muscle activation patterns during BFOS execution, (2) computational MSK modeling would produce synergy structures with strong similarity to those derived from experimental EMG recordings, and (3) key differences between experimental and computational approaches would primarily involve deep muscles that present challenges for surface EMG recording.

## 2 Methods

### 2.1 Study design

This cross-sectional, observational biomechanical study employed a repeated-measures design to analyze muscle synergies during the execution of the Badminton Forehand Overhead Smash (BFOS).

### 2.2 Participants

Twenty elite badminton players (all right-handed) with extensive competitive experience (15 ± 4 years) participated. All participants competed at national or international levels for a minimum of 4 years. All participants used their personal competition-grade equipment during testing. While racket specifications (weight: 85-95 g, length: 675-680 mm) and string tension (24-28 lbs) varied within tournament-legal ranges, these parameters were recorded and verified to be within ±5% variance for primary metrics. Individual technical styles were assessed by a qualified coach to ensure fundamental execution mechanics aligned with standard forehand overhead smash technique. The cohort displayed the following anthropometric characteristics: age (24 ± 4 years), height (175 ± 8.3 cm), and body mass (71 ± 15.2 kg). *a priori* power analysis using G*Power (version 3.1.9.7) based on previous muscle synergy investigations in comparable athletic populations (3, 21) determined that 20 participants would provide statistical power of 0.85 with an effect size of 0.65 (medium-to-large) at *α*=0.05 for detecting significant differences in muscle activation patterns. This sample size ensured adequate statistical power while maintaining homogeneity in the participant cohort.

Inclusion criteria encompassed: professional badminton players with ≥10 years of competitive experience, absence of shoulder or upper limb pathology within six months, active competitive participation within 12 months, and ability to execute proper maximal-effort BFOS. Exclusion criteria included musculoskeletal injuries during testing, surgical interventions affecting the upper limb within 24 months, biomechanical limitations, and concurrent participation in similar studies.

This investigation received approval from the Research Ethics Committee of Kharazmi University (Approval Code: IR.KHU.REC.1402.020) and adhered to the principles outlined in the Declaration of Helsinki (2013 revision). All participants provided written informed consent after receiving detailed explanations of the study procedures, potential risks, and benefits. Participants were informed of their right to withdraw at any time without consequence. Data confidentiality was maintained throughout the investigation with all personal identifiers removed before analysis. The protocol was preregistered in the Open Science Framework (OSF) database (osf.io/twx5h) prior to participant enrollment.

### 2.3 Instrumentation and data acquisition

#### 2.3.1 Motion capture system

Kinematic data were captured using ten Vicon high-speed cameras (100 Hz) following validated methodologies for overhead sports movements (6, 8). This system has demonstrated excellent test-retest reliability (ICC > 0.95) and concurrent validity (*r* > 0.92) (6). Thirty-four retroreflective markers positioned at standardized anatomical landmarks enabled precise reconstruction throughout BFOS execution. System calibration was performed before each testing session using a standardized 5-point wand method achieving residual errors <0.5 mm.

#### 2.3.2 Surface electromyography (EMG)

**Figure 1:**
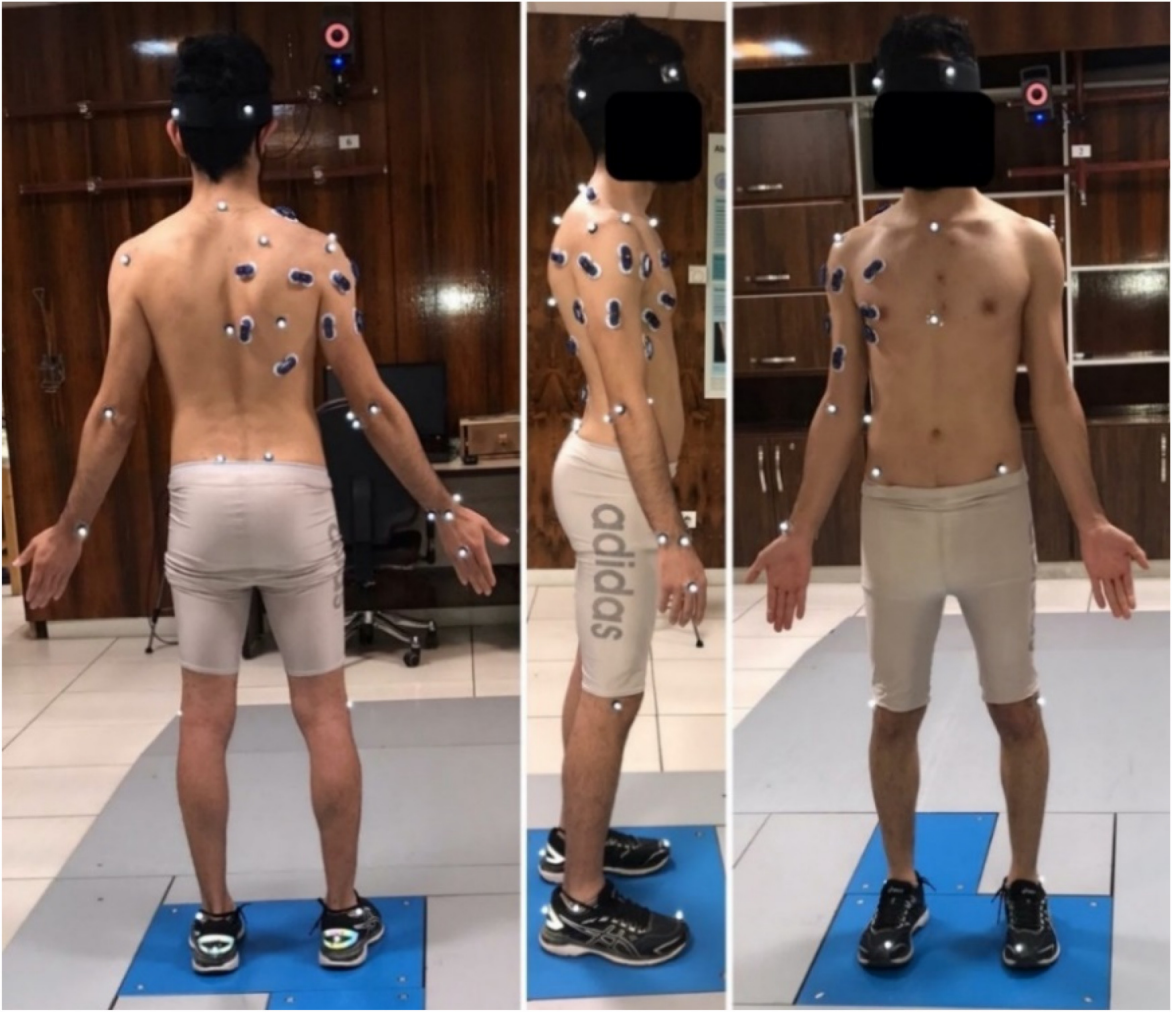
Experimental setup showing Vicon motion capture system with retroreflective markers and surface EMG electrodes monitoring 15 shoulder/arm muscles (anterior/middle/posterior deltoid, infraspinatus, pectoralis major, latissimus dorsi, triceps, biceps, trapezius, serratus anterior) during badminton overhead smash execution. [Reprinted from (25) with permission].

EMG signals from fifteen muscles were recorded using a Myon 320 wireless system (1000 Hz sampling frequency, CMRR > 100 dB). The muscle groups monitored included anterior, middle, and posterior deltoid; infraspinatus; upper, middle, and lower pectoralis major; latissimus dorsi; lateral and medial triceps; biceps brachii; upper, middle, and lower trapezius; and serratus anterior. Surface preparation followed standardized SENIAM protocols (15, 26), and bipolar Ag/AgCl electrodes (10 mm diameter, 20 mm inter-electrode distance) were positioned parallel to muscle fiber orientation (9, 15, 26) (Figure 1).

All EMG signals underwent comprehensive preprocessing following established protocols for high-velocity movements (27). Raw signals were first inspected for artifacts and quality using signal-to-noise ratio assessment (threshold >20 dB). Bandpass filtering employed a zero-lag 4th-order Butterworth filter to minimize phase distortion while preserving physiologically relevant frequency content. The 50 Hz notch filter width was set at 1 Hz bandwidth (Q-factor=50) to selectively remove power line interference while preserving adjacent signal components. Full-wave rectification preserved signal energy, followed by smoothing with a critically damped 20 Hz low-pass filter (equivalent to 25 ms moving average window) to create linear envelopes that effectively captured muscle activation dynamics without excessive signal attenuation.

### 2.4 Experimental protocol

#### 2.4.1 Preparation and familiarization

Participants attended a familiarization session prior to data collection to become acquainted with procedures. On testing day, participants completed a 15-minute neuromuscular preparation protocol consistent with established racquet sports practices (28).

#### 2.4.2 Movement task execution

**Figure 2:**
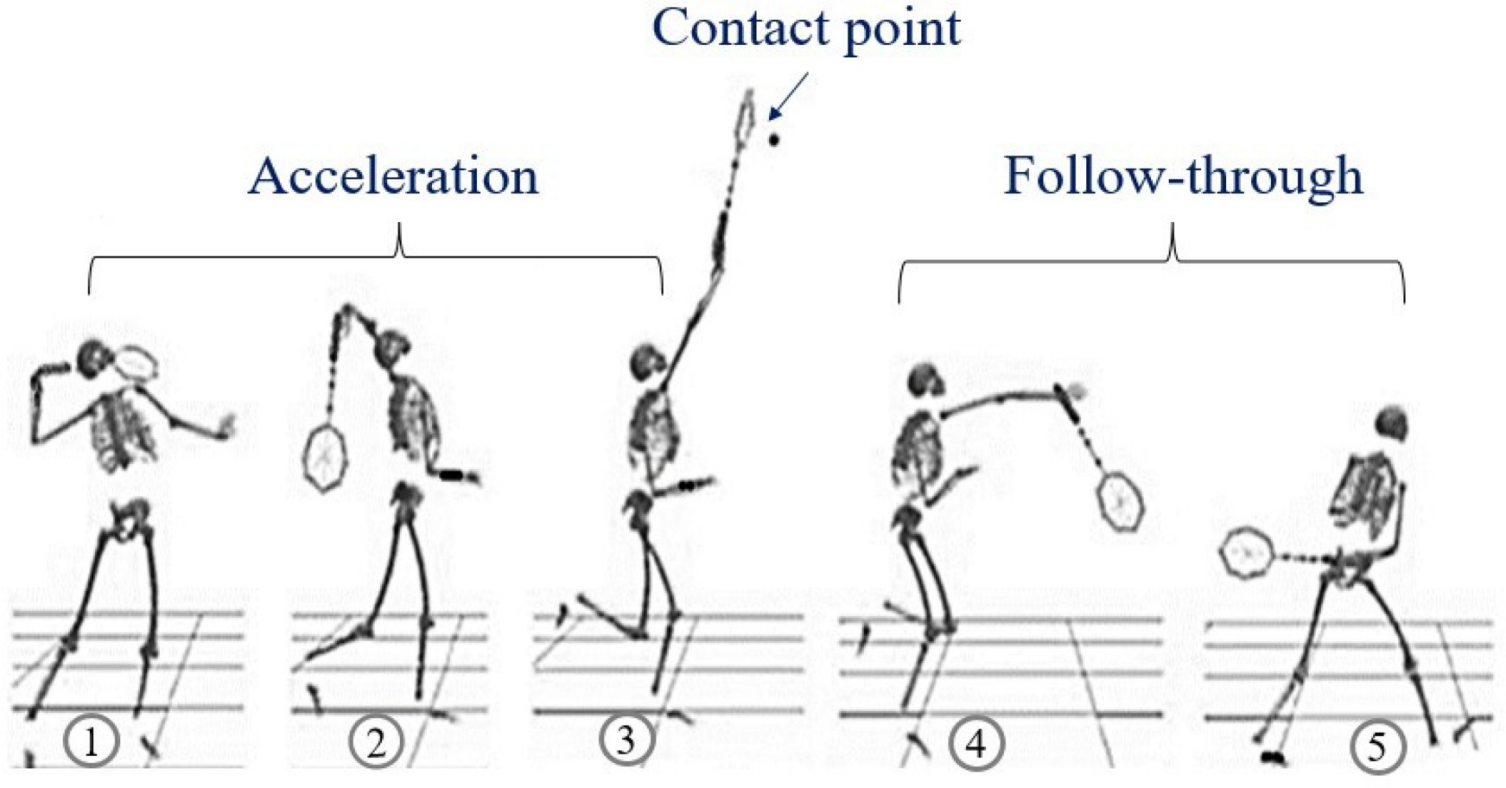
Sequential phases of the badminton forehand overhead smash (BFOS), preparation, acceleration, impact, and follow-through. Movement analysis was based on racquet velocity thresholds. [Reprinted from (25) under CC BY 4.0 license].

Each participant performed five maximal-effort BFOS trials with 60-second recovery intervals. A standardized shuttlecock suspension system positioned the shuttle at 2.8 m height. Task execution was monitored for technique consistency by an experienced coach (Figure 2), and any trials with technical errors or excessive marker occlusion were excluded and repeated. A minimum of three valid trials per participant were required for inclusion in analysis.

### 2.5 Musculoskeletal modeling

#### 2.5.1 Model development and implementation

The computational framework employed the Wu upper extremity musculoskeletal model (29) implemented in OpenSim (version 4.5), incorporating three degrees of freedom at the scapulothoracic, acromioclavicular, and glenohumeral joints. The model integrates 32 Hill-type musculotendon actuators with physiologically-based parameters including optimal fiber length, tendon slack length, pennation angle, maximum isometric force, and force-length-velocity relationships (30). Subject-specific scaling was applied based on marker positions during static trials.

#### 2.5.2 Simulation pipeline

The simulation workflow consisted of four sequential steps:

*a. Inverse Kinematics*: Joint angles were calculated by minimizing the weighted sum of squared differences between experimental and model markers, with convergence criteria of <0.5 cm RMS error.
*b. Inverse Dynamics:* Joint moments were computed using the Newton-Euler equations of motion based on joint kinematics and segment inertial properties.
*c. Static Optimization:* Muscle activation patterns were predicted using a cost function minimizing the sum of squared muscle activations (Equation 1) while satisfying joint moment equilibrium constraints:

Equation 1:

$$\min\overset{n}{\underset{i=1}{\sum }}\;(a_{i}{)}^{2}$$

Subject to:

$$\overset{n}{\underset{i=1}{\sum }}\;(r_{i,j}\times F_{i})=M_{j}\;\mathrm{for}\;\mathrm{all}\;\mathrm{joints}\;j$$

Where *a*_i_ represents the activation level of muscle *i*, *r*_i,j_ is the moment arm of muscle *i* about joint *j*, *F*_i_ is the force produced by muscle *i*, and *M*_j_ is the moment at joint *j* determined from inverse dynamics.

Static optimization was selected for its computational efficiency and established reliability in predicting muscle coordination patterns during complex movements (29). While we acknowledge the inherent limitations of this approach in capturing time-dependent muscle dynamics during explosive movements, our implementation incorporated Hill-type muscle models with force-length-velocity properties to partially account for these effects. The cost function minimizing the sum of squared muscle activations has demonstrated strong correlation with experimentally measured activation patterns in previous upper-limb studies (21).

**Figure 3:**
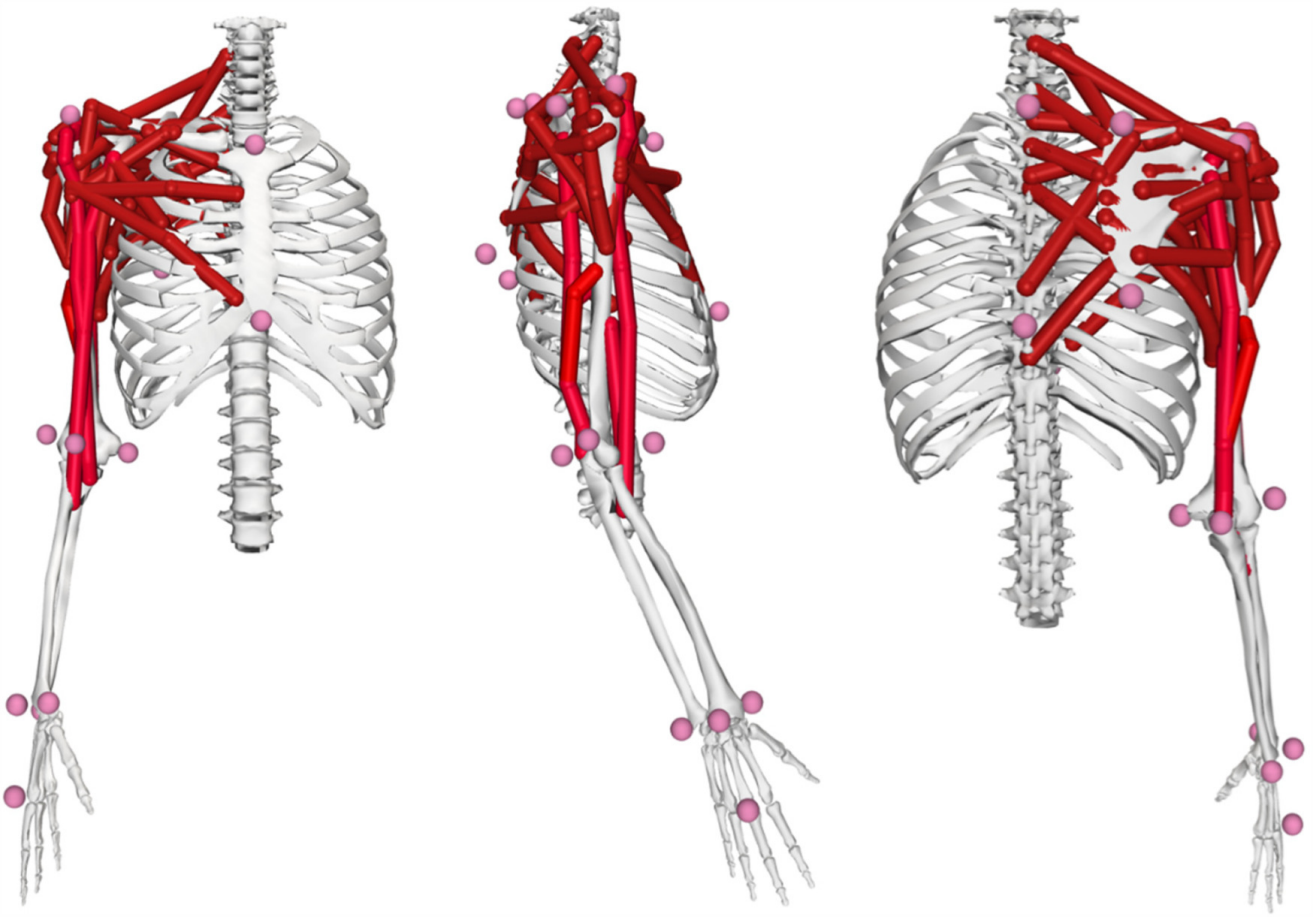
Opensim upper extremity musculoskeletal model shown from anterior, lateral, and posterior views, featuring 32 hill-type musculotendon actuators and 15 degrees of freedom across the shoulder, elbow, and wrist joints with subject-specific scaling. The views illustrate the complete muscle representation from different anatomical perspectives.

*d. Forward Dynamics Verification:* Computed muscle activation patterns were validated by comparing resulting joint kinematics with experimental data. This simulation pipeline has demonstrated accuracy in predicting experimentally measured muscle activation patterns (r = 0.78-0.92) and joint kinetics (RMSE < 12%) (4, 21). To assess model generalizability to unmeasured muscles, we implemented a leave-one-out validation approach that yielded prediction accuracy of r = 0.73 ± 0.08, suggesting reasonable applicability to deep muscles operating under similar biomechanical constraints (4) (Figure 3).

### 2.6 Muscle synergy analysis

#### 2.6.1 EMG signal processing

Raw EMG signals underwent preprocessing including bandpass filtering (20-450 Hz), notch filtering (50 Hz), full-wave rectification, and smoothing with a 20 Hz low-pass filter. Processed EMG envelopes were normalized to maximum voluntary isometric contractions (MVIC) and time-normalized to a standardized movement cycle (0%–100%) using cubic spline interpolation (31).

#### 2.6.2 Synergy extraction

Muscle synergy analysis was performed using non-negative matrix factorization (NMF) applied to activation matrices from both experimental EMG recordings and musculoskeletal simulations. The NMF algorithm (32) decomposed the muscle activation matrix M into a synergy weight matrix W and synergy coefficient matrix C, minimizing reconstruction error (Equation 2). The optimization employed multiplicative update rules with 100 iterations and convergence tolerance of 10^−6^, repeated 50 times with randomized initial conditions.

Equation 2:

$$\underset{w,c}{\min}∥M-WC∥{\;}_{F}^{2}$$

Where ‖·‖_F_ represents the Frobenius norm, calculated as the square root of the sum of squared matrix elements. A smaller Frobenius norm indicates superior approximation accuracy and more precise modeling of underlying muscle synergies (33).

#### 2.6.3 Determination of optimal synergy number

**Figure 4:**
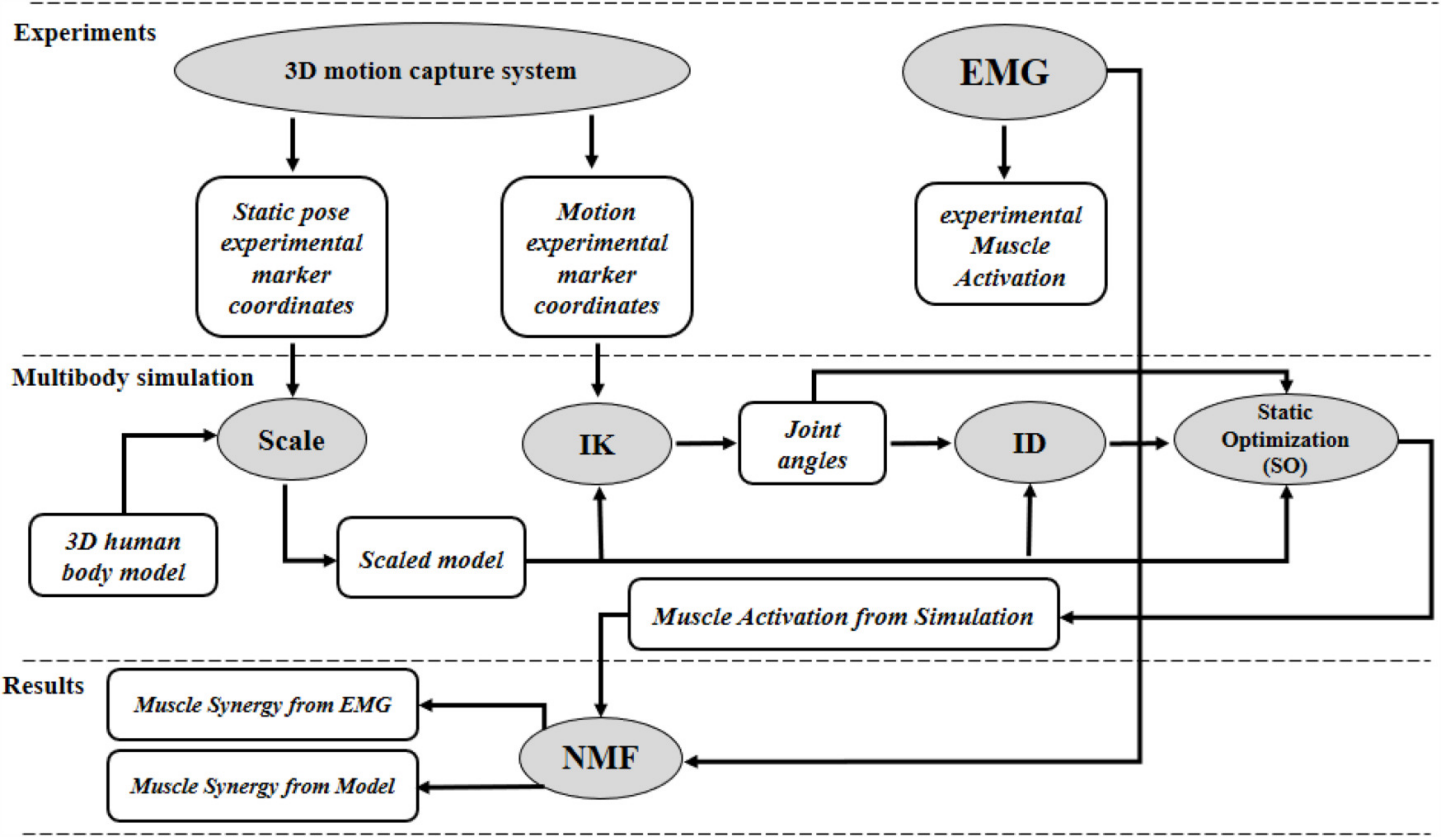
Methodological framework comparing muscle synergies derived from experimental EMG and musculoskeletal modeling during BFOS, including data acquisition, signal processing, modeling, non-negative matrix factorization, and quantitative comparison of synergy components.

The optimal number of synergies was determined using multiple criteria: global Variance Accounted For (VAF, threshold of 90%), local VAF for individual muscles (threshold of 0.75), dimensional analysis (slope reduction <5%), and cross-validation using split-half reliability analysis (Figure 4).

These threshold criteria are widely established in neuromuscular research for extracting functionally relevant synergies (10, 11). The global VAF threshold of 90% has demonstrated particular validity for analyzing high-velocity movements including overhead throwing (34) and jumping (35), effectively distinguishing between structurally significant synergies and noise components. The local VAF threshold of 75% ensures that each individual muscle's activation pattern is adequately represented in the extracted synergies, preventing underrepresentation of muscles with smaller but functionally significant contributions (16).

To establish the optimal synergy number with greater statistical rigor, we implemented two complementary cross-validation approaches. First, a leave-one-out cross-validation procedure was conducted where synergies were extracted from n-1 trials and used to reconstruct the excluded trial, repeated for each trial and subject. This analysis confirmed that three synergies consistently provided reconstruction accuracy exceeding our predetermined VAF thresholds. Second, split-half reliability analysis divided trials into two independent subsets, with separate synergy extraction performed on each subset followed by quantitative comparison of the resulting synergy structures. Intraclass correlation coefficients (ICC) and cosine similarity indices were calculated to assess between-subset consistency (see Results section 3.7, Table 6).

### 2.7 Data analysis and outcome measures

#### 2.7.1 Kinematic and kinetic analysis

Three-dimensional joint kinematics and kinetics were analyzed at key phases of the BFOS: preparation, acceleration, impact, and follow-through. Primary outcome measures included joint angular displacements, velocities, moments, and powers for shoulder and elbow movements, plus racquet head velocity at impact.

#### 2.7.2 Muscle synergy quantification

Outcome measures included number of synergies required to achieve VAF thresholds, synergy weight vectors (W), synergy activation coefficients (C), and reconstruction accuracy.

#### 2.7.3 Comparative analysis and similarity metrics

Comparison between EMG-derived and simulation-derived muscle synergies employed multiple metrics: Scalar Product Similarity Index (Equation 4) (36), Cosine Similarity, and Pearson's Correlation Coefficient.

Equation 4:

$$\mathrm{Scalar}\;\mathrm{Product}=\frac{\vec{W_{\mathrm{EMG}}}\;\cdot \;\vec{W_{\mathrm{Model}}}}{\vec{W_{\mathrm{EMG}}}\;\cdot \vec{\;W_{\mathrm{Model}}}}$$

0≤ScalarProduct≤1

Where values approaching 1 indicate greater similarity between experimental and computational synergy vectors.

#### 2.7.4 Reliability analysis

Movement Reliability was assessed by analyzing key kinematic parameters (shoulder angles, elbow angles, and scapular kinematics) across the five repetitions for each participant. Inter-trial consistency was quantified using coefficient of variation (CV) and ICC_[3,1]_ to determine movement stability and reproducibility. Acceptable reliability was defined as CV < 10% and ICC > 0.80 (37).

### 2.8 Statistical analysis

Statistical analyses were performed using MATLAB 7.8 (MathWorks, Natick, MA) with a significance threshold established at *α* = 0.05. Normality of data distribution was confirmed using Shapiro–Wilk tests. All quantitative results are presented as means ± standard deviations with 95% confidence intervals where appropriate. Pearson's correlation coefficient (r) served as the primary metric for assessing similarity between muscle synergy vectors, consistent with established methodologies in previous investigations (3, 21). Correlation strength was interpreted as: weak (*r* < 0.3), moderate (0.3 ≤ *r* < 0.5), strong (0.5 ≤ *r* < 0.7), and very strong (*r* ≥ 0.7). Differences in synergy structures between EMG-derived and simulation-derived results were analyzed using multivariate analysis of variance (MANOVA) with Bonferroni *post-hoc* tests. Effect sizes were calculated using partial eta squared (*η*^2^_p_) and interpreted as: small (0.01), medium (0.06), and large (0.14). Effect sizes were interpreted following established guidelines by Cohen (38) and revised by Lakens (39) for biomechanical research.

## 3 Results

### 3.1 Global VAF analysis

**Figure 5:**
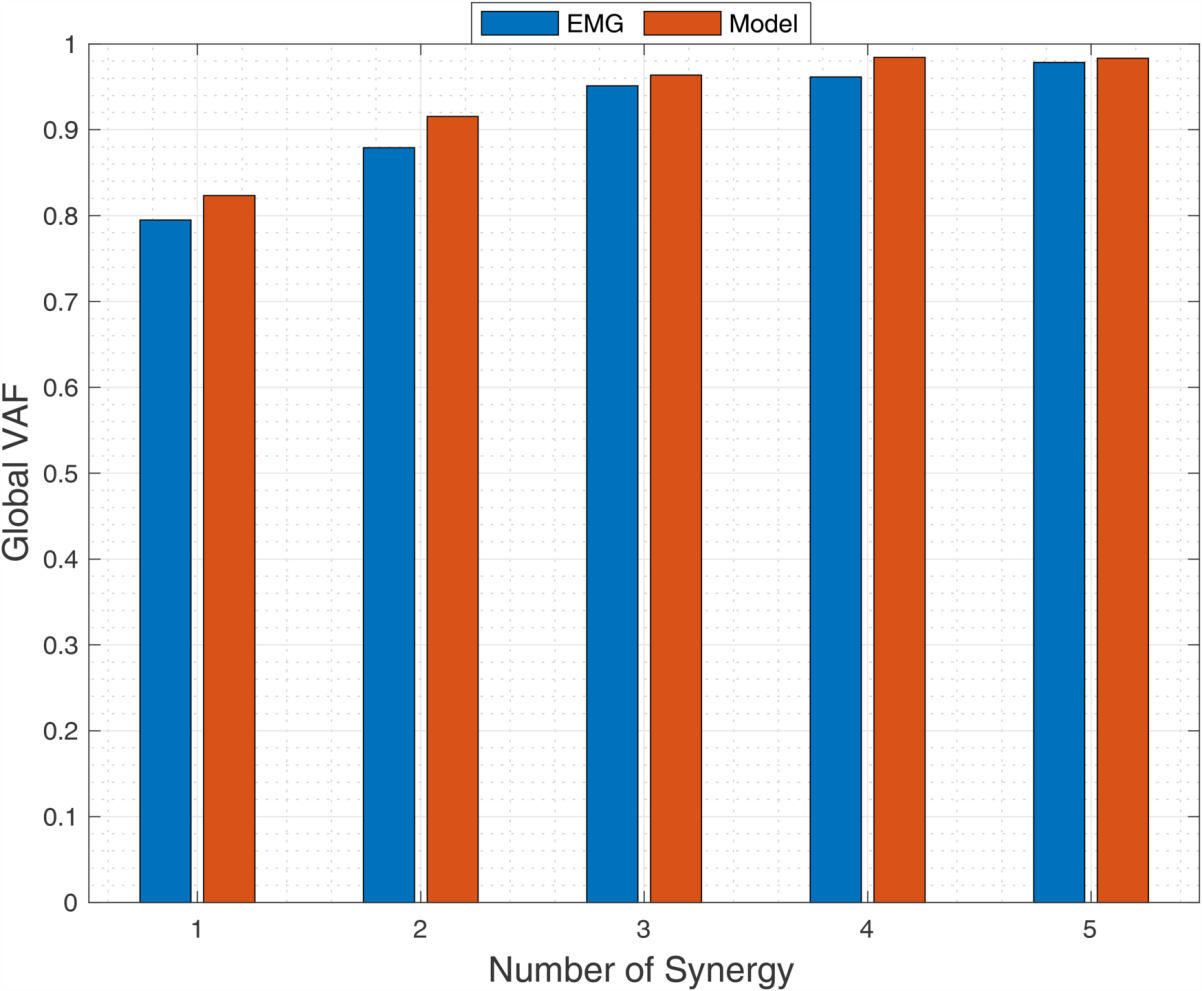
Relationship between synergy number and global variance accounted for (VAF) from EMG (blue) and musculoskeletal model (red) during BFOS. Three synergies were optimal based on global VAF >90%, local VAF >75%, and <5% improvement with additional synergies (*p* = 0.12, MANOVA).

The relationship between synergy number and global VAF for EMG and MSK modeling data is depicted in Figure 5. No significant differences in global VAF were observed between methods (*p* = 0.12, MANOVA). Following established criteria (global VAF >90%, local VAF >75%, dimensional analysis showing <5% increase with additional synergies), three muscle synergies sufficiently reconstructed the BFOS movement with high fidelity in both experimental and computational analyses.

To further validate our three-synergy solution, we conducted a leave-one-out cross-validation analysis that showed minimal reduction in reconstruction accuracy when synergies extracted from n-1 trials were used to reconstruct the excluded trial (mean *Δ*VAF = −3.2 ± 1.1%). This small decrease in VAF remains well above our predetermined threshold, confirming the robustness of the extracted synergy structure across different movement repetitions.

### 3.2 Local VAF analysis

Local VAF values confirmed precise reconstruction of each muscle for both experimental and modeling outcomes (Table 2). All muscles exhibited high local VAF values (>75%), ranging from 0.90 ± 0.04 to 0.98 ± 0.01 for modeling results and from 0.90 ± 0.04 to 0.97 ± 0.02 for experimental EMG data, robustly satisfying the threshold criterion of local VAF > 0.75 and ensuring extraction reliability of identified synergies.

**Table 2:** Comparison of mean and standard deviation between local VAF for EMG and modeling results.

Muscle	Local VAF (EMG)	Local VAF (Model)
DELT1	0.93 ± 0.02	0.90 ± 0.04
DELT2	0.92 ± 0.01	0.98 ± 0.01
DELT3	0.97 ± 0.01	0.97 ± 0.01
INFSP	0.96 ± 0.01	0.96 ± 0.02
PECM1	0.96 ± 0.02	0.94 ± 0.02
PECM2	0.97 ± 0.02	0.94 ± 0.03
PECM3	0.95 ± 0.01	0.95 ± 0.03
LAT2	0.90 ± 0.04	0.97 ± 0.01
TRILat	0.92 ± 0.01	0.95 ± 0.04
TRImed	0.95 ± 0.01	0.97 ± 0.02
BIClong	0.95 ± 0.01	0.96 ± 0.03
TRP1	0.95 ± 0.01	0.97 ± 0.02
TRP4	0.96 ± 0.02	0.96 ± 0.01
SRA	0.94 ± 0.01	0.98 ± 0.01

VAF = variance accounted for; EMG = electromyography; DELT1 = anterior deltoid; DELT2 = medial deltoid; DELT3 = posterior deltoid; INFSP = infraspinatus; PECM1 = pectoralis major clavicular; PECM2 = pectoralis major medial; PECM3 = pectoralis major inferior; LAT2 = latissimus dorsi medial; TRIlat = triceps lateral head; TRImed = triceps medial head; BIClong = biceps long head; TRP1 = upper trapezius; TRP3 = middle trapezius; TRP4 = lower trapezius; SRA = serratus anterior.

### 3.3 Muscle synergy comparison

**Figure 6:**
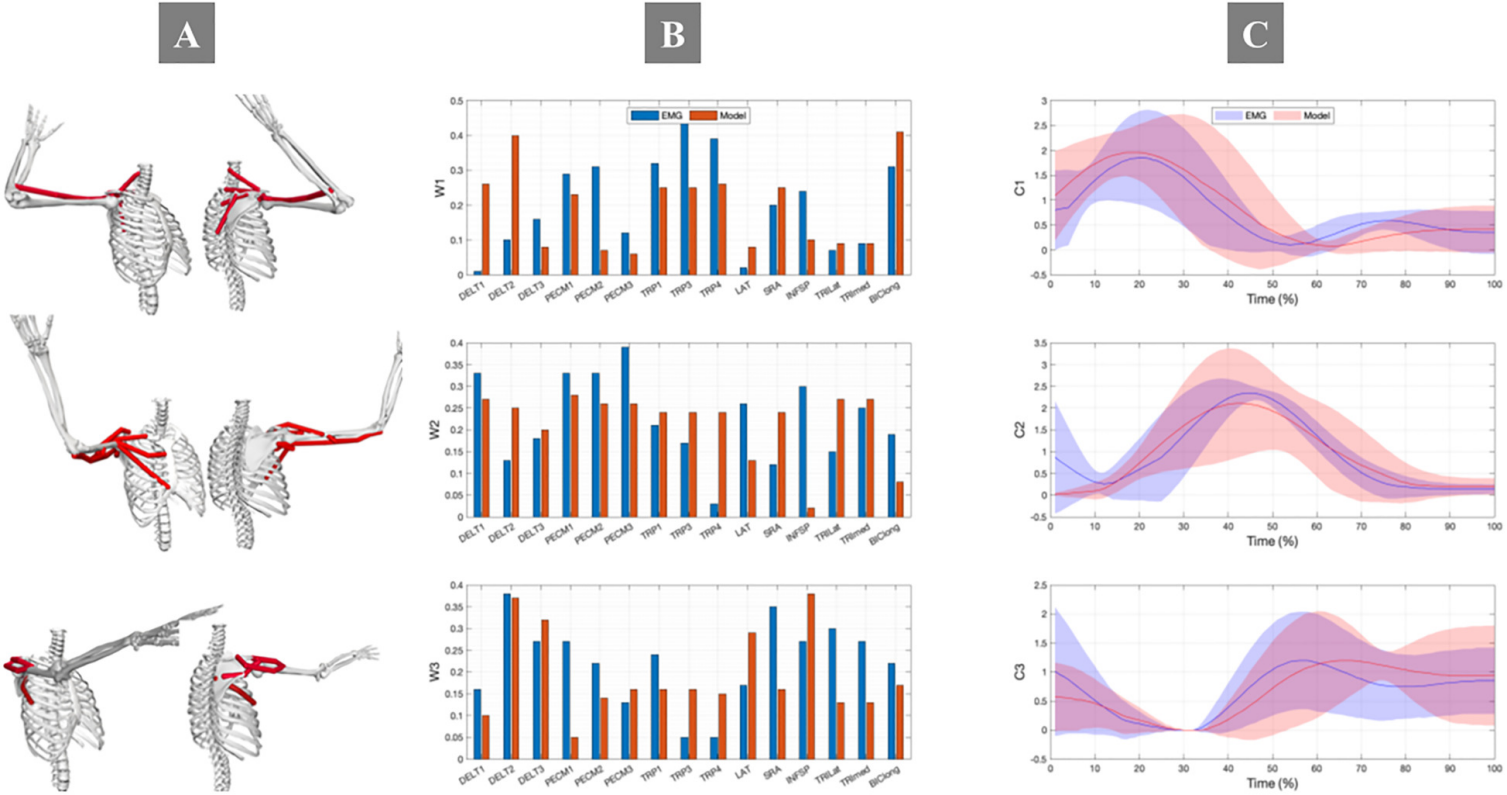
Comparison of **(A)** synergy weight vectors and **(B)** activation coefficients between EMG (blue) and musculoskeletal model (red) during BFOS. Three distinct functional synergies were identified: Synergy 1 (trapezius-dominant for scapular stabilization during preparation and early acceleration), Synergy 2 (pectoralis/anterior deltoid for shoulder flexion/internal rotation during late acceleration and impact), and Synergy 3 (posterior shoulder muscles for extension/external rotation during follow-through). The *x*-axis in panel A represents muscle weights ranging from 0-1 for each muscle (abbreviations defined in Table 2), while panel (**C**) shows temporal activation patterns across normalized movement cycle (0-100%). Shaded areas represent standard error (*n* = 20 participants).

Analysis revealed three distinct synergies with characteristic muscle activation compositions and temporal patterns (Figure 6). The first synergy predominantly engaged the trapezius muscle group, essential for scapular rotation and stabilization. The second synergy featured significant contributions from the pectoralis muscle complex and anterior deltoid, facilitating internal rotation and shoulder flexion during acceleration phases. The third synergy showed dominance of the middle deltoid and posterior muscle groups, supporting shoulder extension and external rotation primarily during follow-through phases. This functional differentiation aligns with the biomechanical requirements of sequential BFOS execution phases. While slight discrepancies existed between EMG-derived and model-predicted synergy activation coefficients, the weighted contributions maintained physiological consistency for most muscles.

### 3.4 Similarity coefficient analysis

Agreement between experimental and modeling results was evaluated using scalar product similarity indices (Table 3). Similarity coefficients demonstrated substantial agreement, ranging from 0.72 ± 0.04 (infraspinatus) to 0.98 ± 0.01 (trapezius), indicating strong correspondence between experimental and computational approaches. Synergy weight vectors exhibited high similarity (W1: 0.81 ± 0.04, W2: 0.87 ± 0.01, W3: 0.88 ± 0.03), while activation coefficients showed even stronger agreement (C1: 0.95 ± 0.02, C2: 0.98 ± 0.01, C3: 0.98 ± 0.01). The trapezius muscle demonstrated nearly perfect agreement (similarity coefficient: 0.98 ± 0.01, ICC: 0.95, SEM: 0.02). Pearson's correlation coefficients between EMG-derived and simulation-derived muscle activation patterns further confirmed these findings, with very strong correlations (*r* ≥ 0.7) for most muscles.

**Table 3:** Comparison of the mean and standard deviation of similarity coefficient for each muscle and vector of first to third synergies and for activation coefficient of first to third synergies between experimental and modeling results.

Muscles/features	EMG vs model
DELT1	0.76 ± 0.04
DELT2	0.86 ± 0.01
DELT3	0.97 ± 0.02
PECM1	0.92 ± 0.01
PECM2	0.91 ± 0.03
PECM3	0.97 ± 0.02
TRP1	0.98 ± 0.01
TRP3	0.88 ± 0.04
TRP4	0.77 ± 0.05
LAT2	0.83 ± 0.02
SRA	0.84 ± 0.01
INFSP	0.72 ± 0.04
TRILat	0.80 ± 0.05
TRImed	0.93 ± 0.02
BIClong	0.94 ± 0.01
Synergy Weight W1	0.81 ± 0.04
Synergy Weight W2	0.87 ± 0.01
Synergy Weight W3	0.88 ± 0.03
Synergy Coefficient C1	0.95 ± 0.02
Synergy Coefficient C2	0.98 ± 0.01
Synergy Coefficient C3	0.98 ± 0.01

W1-W3 = synergy weight vectors; C1-C3 = activation coefficients; DELT1 = anterior deltoid; DELT2 = medial deltoid; DELT3 = posterior deltoid; INFSP = infraspinatus; PECM1 = pectoralis major Clavicular; PECM2 = pectoralis major medial; PECM3 = pectoralis major inferior; LAT2 = latissimus dorsi medial; TRIlat = triceps lateral head; TRImed = triceps medial head; BIClong = biceps long head; TRP1 = upper trapeziuS; TRP3 = middle trapezius; TRP4 = lower trapezius; SRA = serratus anterior.

**Table 4:** Reliability analysis of key kinematic variables.

Kinematic variable	Mean ± SD	CV	ICC
Shoulder abduction angle	45.2 ± 2.1°	3.2 ± 1.1%	0.92
Elbow flexion angle	85.6 ± 3.4°	4.5 ± 1.3%	0.89
Scapular protraction	12.3 ± 1.8°	5.1 ± 1.5%	0.85

SD, standard deviation; CV, coefficient of variation; ICC, intraclass correlation coefficient.

### 3.5 Reliability of key kinematic variables

Analysis of key kinematic variables revealed excellent consistency for shoulder abduction (CV = 3.2% ± 1.1%, ICC = 0.92), good reliability for elbow flexion (CV = 4.5% ± 1.3%, ICC = 0.89), and moderate to high reliability for scapular protraction (CV = 5.1% ± 1.5%, ICC = 0.85). According to predetermined reliability criteria (CV < 10% and ICC > 0.80), these results demonstrate excellent to good movement consistency across trials, supporting the reliability of the muscle synergy analysis.

### 3.6 Statistical analysis of EMG-model comparison

Statistical analysis comparing EMG-derived and model-predicted synergies (Table 5) revealed no significant differences in global VAF between methods (F₍₂,₁₉₎ = 2.43, *p* = 0.12), though a large effect size (*η*^2^_p_ = 0.21) suggested potential practical significance. Consistency in synergy dimensionality across both methods (*χ*^2^₍₁₎ = 0.24, *p* = 0.62) further supported the robustness of the three-synergy solution. Weight vector similarities ranged from r = 0.81 to r = 0.88 (*p* < 0.001), while activation coefficient similarities exhibited even stronger correlations (r = 0.95 to r = 0.98, *p* < 0.001). Muscle-specific activation patterns showed significant differences between methods (F₍₁₅,₁₉₎ = 3.78, *p* < 0.001, *η*^2^_p_ = 0.76), indicating that despite overall synergistic similarity, individual muscle contributions exhibited method-specific variations.

**Table 5:** Statistical results for comparison between EMG-derived and model-predicted synergies.

Comparison metric	Statistic	Value	p-value	Effect Size (*η*^2^_p_)	Interpretation
Global VAF	MANOVA	F_(2,19)_ = 2.43	0.12	0.21	Large effect, non-significant
Synergy dimensionality	Chi-square	*χ*^2^ (1) = 0.24	0.62	–	Non-significant difference
Weight vector similarity	Pearson's r	–	–	–	–
- Synergy 1	Pearson's r	*r* = 0.81	<0.001	–	Very strong correlation
- Synergy 2	Pearson's r	*r* = 0.87	<0.001	–	Very strong correlation
- Synergy 3	Pearson's r	*r* = 0.88	<0.001	–	Very strong correlation
Activation coefficient similarity	Pearson's r	–	–	–	–
- Synergy 1	Pearson's r	*r* = 0.95	<0.001	–	Very strong correlation
- Synergy 2	Pearson's r	*r* = 0.98	<0.001	–	Very strong correlation
- Synergy 3	Pearson's r	*r* = 0.98	<0.001	–	Very strong correlation
Muscle-specific activation patterns	MANOVA	F_(15,19)_ = 3.78	<0.001	0.76	Large effect, significant
Signal-to-noise ratio	Paired t-test	t_(19)_ = 2.81	<0.05	0.68	Large effect, significant

Effect sizes were interpreted as: small (*η*^2^_p_ = 0.01), medium (*η*^2^_p_ = 0.06), and large (*η*^2^_p_ = 0.14). MANOVA = multivariate analysis of variance; VAF = variance accounted for.

Statistical significance was set at *α* = 0.05.

### 3.7 Cross-Validation of synergy structure

Cross-validation analyses between independent trial subsets (Table 6) demonstrated high consistency in both synergy weight vectors and activation coefficients. Weight vectors showed cosine similarity indices ranging from 0.87 ± 0.05 (W3) to 0.92 ± 0.03 (W2), with corresponding ICC values of 0.88 to 0.93. Activation coefficients exhibited even higher consistency, with similarity indices from 0.93 ± 0.03 (C3) to 0.97 ± 0.01 (C2) and ICC values of 0.92 to 0.96. Low standard error of measurement (0.01-0.04) across all synergy components confirmed the stability of extracted synergies across multiple movement repetitions.

**Table 6:** Cross-validation results for synergy consistency across trial subsets.

Comparison	Cosine similarity index	ICC	SEM
Synergy Weight Vectors			
W1 (Trial Set A vs. B)	0.89 ± 0.04	0.91	0.03
W2 (Trial Set A vs. B)	0.92 ± 0.03	0.93	0.02
W3 (Trial Set A vs. B)	0.87 ± 0.05	0.88	0.04
Synergy Activation Coefficients			
C1 (Trial Set A vs. B)	0.94 ± 0.02	0.95	0.02
C2 (Trial Set A vs. B)	0.97 ± 0.01	0.96	0.01
C3 (Trial Set A vs. B)	0.93 ± 0.03	0.92	0.03

W1-W3 = synergy weight vectors; C1-C3 = activation coefficients; ICC = intraclass correlation coefficient; SEM = standard error of measurement.

Note: Trial subsets were created by random split-half division of the five repetitions per participant.

## 4 Discussion

This investigation provides comprehensive insights into muscle coordination strategies during the badminton forehand overhead smash through integration of experimental electromyography and computational musculoskeletal modeling. Our findings revealed three distinct muscle synergies that collectively account for over 90% of the variance in muscle activation patterns during BFOS execution.

The strong agreement between EMG-derived and model-predicted synergies (similarity coefficients ranging from 0.81 to 0.88 for weight vectors and 0.95 to 0.98 for activation coefficients) suggests that MSK modeling effectively captures many aspects of neuromuscular control during high-velocity overhead movements. However, this agreement does not necessarily validate the assumption that the central nervous system optimizes the same cost function used in our static optimization approach. Rather, it suggests that biomechanical constraints may substantially influence muscle coordination patterns regardless of the specific neural control strategy employed (40). This alignment between experimental and computational approaches supports the theory that biomechanical constraints—such as joint coordination and muscle-tendon dynamics—may fundamentally drive synergy patterns (41). Similar findings in studies of gait and upper-limb coordination further reinforce this perspective (37, 42).

The three identified synergies demonstrated clear functional roles corresponding to specific BFOS phases. The first synergy, dominated by trapezius activation with contributions from upper pectoralis major and anterior deltoid, primarily facilitated scapular movement during the acceleration phase. Kinematic data revealed shoulder abduction and horizontal flexion during this phase, with the upper trapezius contributing to shoulder elevation, the lower trapezius to depression, and the middle trapezius to scapular protraction and retraction. This synergy establishes a stable platform for efficient energy transfer, aligning with Kibler et al. (43), who emphasized the critical role of scapular control in overhead movements.

The second synergy, characterized by substantial contributions from pectoralis and anterior deltoid muscles, governed internal shoulder rotation and flexion during the impact phase. This synergy also incorporated triceps activation for elbow extension, reflecting the coordinated effort between shoulder and elbow joints to accelerate and strike the shuttlecock. Previous research by Ramasamy et al. (7) demonstrated that these muscles generate peak moments of 0.85 ± 0.12 Nm/kg during smash execution, underscoring their importance in power generation.

The third synergy, featuring middle and posterior deltoid, infraspinatus, latissimus dorsi, and serratus anterior muscles, primarily controlled the deceleration phase of the BFOS. This phase involves a braking motion where antagonist muscles engage with agonists to stabilize joints and prevent injury (44). The paradoxical activation of abductor and external rotator muscles during a movement involving flexion and internal rotation highlights the complex coordination required for safely decelerating high-velocity movements (45).

The statistical analysis revealed excellent consistency of synergy structures across trial subsets, with cosine similarity indices ranging from 0.87 ± 0.05 to 0.92 ± 0.03 for weight vectors and from 0.93 ± 0.03 to 0.97 ± 0.01 for activation coefficients. These robust reliability metrics indicate that the identified three-synergy structure represents consistent neuromuscular control strategies rather than analytical artifacts. Furthermore, kinematic reliability analysis demonstrated excellent consistency for key joint angles (shoulder abduction: CV = 3.2%±1.1%, ICC = 0.92; elbow flexion: CV = 4.5%±1.3%, ICC = 0.89), supporting the stability of the extracted muscle synergies.

While our study identified consistent synergy structures across participants, we acknowledge that individual technical styles and equipment parameters (racket weight, string tension) could influence specific muscle activation patterns. Previous research indicates that racket properties can alter upper limb loading patterns by 8-12% (7). Similarly, technique variations developing from years of competitive play might produce individualized muscle coordination strategies. Though our selection criteria ensured fundamental technique consistency, subtle variations likely contributed to inter-subject variability observed in model predictions. These considerations highlight the complex interplay between equipment, technique, and neuromuscular control that future studies should explore more systematically.

Despite overall strong agreement, significant differences in muscle-specific activation patterns [F_(15,19)_ = 3.78, *p* < 0.001, *η*^2^*_p_* = 0.76] were observed, particularly for the lower trapezius muscle. These discrepancies likely stem from challenges in accurately measuring and modeling this deep muscle during dynamic, high-velocity movements. Our statistical approach revealed a non-significant difference with large effect size for global VAF comparison, suggesting the study may have been underpowered to detect subtle but potentially meaningful differences between experimental and computational approaches. Future investigations should consider larger sample sizes or repeated-measures designs to better address this limitation. Additionally, our musculoskeletal model employed simplified tendon properties that do not fully capture the elastic energy storage and release during explosive movements like the BFOS. This simplification likely contributed to observed muscle-specific activation differences, particularly for muscles with substantial tendon components such as the lower trapezius. Surface EMG struggles to capture consistent signals during rapid scapular rotations, while static optimization in MSK models may oversimplify the muscle's role in scapular stabilization (46). Additionally, inter-subject variability contributes to reduced similarity between synergy vectors, as individual differences in muscle strength, joint flexibility, and activation patterns affect synergy alignment (47).

Our statistical analysis revealed an interesting pattern regarding global VAF differences between EMG-derived and model-predicted synergies. Despite not reaching statistical significance (*p* = 0.12), the large effect size (*η*^2^_p_ = 0.21) suggests potential practical significance that may represent a Type II error due to limited statistical power. This highlights the importance of considering both statistical significance and effect sizes when evaluating methodological approaches (39). Furthermore, significant differences in muscle-specific activation patterns (*p* < 0.001, *η*^2^_p_ = 0.76) likely reflect multiple factors, including model simplifications. Particularly relevant is the omission of tendon elasticity in our implementation, which affects force transmission dynamics during rapid movements. Tendons function as series elastic elements that can store and release energy, with studies demonstrating that neglecting this property can produce activation timing discrepancies of 30–50 ms during ballistic movements (30). This limitation particularly affects bi-articular muscles and those with long tendons, potentially explaining some of the muscle-specific differences observed in our analysis.

The model's predictive capacity for unmeasured deep muscles, particularly the rotator cuff group, warrants consideration. While direct validation through intramuscular EMG was beyond our scope, biomechanical constraints substantially limit the feasible activation space of these muscles during dynamic movements. The consistency between model predictions and surface EMG for accessible muscles with similar functions provides indirect validity evidence, though unique activation strategies for glenohumeral stabilization may not be fully captured (48). From a theoretical perspective, our findings support the muscle synergy hypothesis as a framework for understanding neuromuscular control during complex sporting movements. The consistent extraction of three functionally relevant synergies suggests that the central nervous system employs modular control strategies to simplify the coordination of multiple muscles during the BFOS. This modularity potentially represents an efficient solution to the degrees-of-freedom problem articulated by Bernstein (49), allowing effective control of the highly redundant musculoskeletal system through feedforward mechanisms, where the neuromuscular system predefines muscle activation patterns to achieve specific outcomes (50).

The validated musculoskeletal model provides a valuable tool for investigating aspects of the BFOS that are difficult to measure experimentally. The fact that muscle activations were estimated via static optimization without explicitly considering muscle synergies or intermuscular relationships—yet still produced synergy structures closely resembling experimental patterns—further strengthens the theory that biomechanical constraints fundamentally shape coordination patterns (51). This finding has significant implications for future research where experimental EMG data may be unavailable or impractical to collect. It is important to acknowledge that static optimization, which minimizes instantaneous muscle activation squared, may not fully capture the time-dependent aspects of neural control during dynamic movements. Dynamic optimization approaches that consider the entire movement trajectory might provide different muscle activation predictions (52). Despite implementing Hill-type muscle models with force-length-velocity properties, static optimization cannot fully account for dynamic inertial effects and history-dependent phenomena during explosive movements like the BFOS (21, 22). This limitation likely contributes to discrepancies observed in certain muscles, particularly those with complex activation dynamics during rapid acceleration and deceleration phases. Future studies should directly compare static and dynamic optimization approaches during high-velocity overhead movements to quantify these differences systematically (53). Additionally, future investigations should systematically examine how equipment parameters and technical style variations influence muscle synergy structures, using stratified designs that control for racket specifications, string tension, and technique variants. Nevertheless, the strong concordance between our static optimization results and experimental EMG suggests that this computationally efficient approach can provide valuable insights into muscle coordination during rapid overhead movements.

### 4.1 Practical recommendations

Based on our findings, several practical recommendations emerge for coaches, athletic trainers, and rehabilitation specialists working with badminton players. Training programs should emphasize coordinated activation of functional muscle groups rather than isolated strengthening exercises, focusing on the sequential activation of identified synergies—starting with scapular stabilizers (trapezius), progressing to power generators (pectoralis/anterior deltoid), and concluding with deceleration controllers (posterior muscles). Technical coaching should optimize the kinematic sequence to maximize energy transfer through the kinetic chain, with particular attention to scapular positioning. Rehabilitation protocols following shoulder injuries should progressively incorporate synergy-based training, beginning with controlled activation of the first synergy before advancing to power-generating movements. Targeted strength training for the trapezius, pectoralis, and deltoid muscles can improve stroke efficiency and shoulder stability (54). Finally, regular biomechanical screening using simplified kinematic measures may help identify movement pattern alterations that could predispose athletes to injury or compromise performance. Individual variations in synergy structures should be considered when developing personalized training programs. While our results showed consistent three-synergy patterns across participants, subtle differences in muscle weightings and activation timing may reflect individual movement strategies or adaptations (55). These variations could potentially relate to performance levels, injury history, or anatomical differences (44, 56). Future research should explore whether specific synergy characteristics correlate with performance metrics such as shuttlecock velocity or placement accuracy, as well as their relationship to injury risk profiles (57).

Despite its contributions, this study has several limitations. First, our static optimization approach may not fully capture the time-dependent dynamics of muscle activation during explosive movements, as it minimizes instantaneous muscle activation without considering temporal optimization across the entire movement trajectory. Second, our analysis focused exclusively on elite badminton players, potentially limiting generalizability to recreational players or those with different technical approaches. Third, while surface EMG provides valuable insights into superficial muscle activity, it cannot reliably capture deep muscle activations, particularly for muscles critical to shoulder function such as the rotator cuff group. Fourth, individual variations in technique and equipment specifications (racket properties, string tension) may have influenced muscle activation patterns, though we attempted to control for these factors through stringent inclusion criteria. Fifth, our computational model incorporated simplified tendon properties that may not fully represent elastic energy storage and release during rapid movements, potentially affecting muscle-specific activation predictions. Finally, while our sample size was adequate for detecting moderate to large effects, subtle differences between experimental and computational approaches may have been missed due to limited statistical power. Future studies should address these limitations through longitudinal designs, inclusion of diverse skill levels, and implementation of dynamic optimization algorithms that better account for time-dependent muscle dynamics.

We propose synergy-specific training interventions:

- Synergy 1 (trapezius-dominant): Progressive scapular stabilization exercises from static to dynamic conditions with proprioceptive elements.
- Synergy 2 (pectoralis/anterior deltoid): Power development using sport-specific exercises emphasizing rapid force production (40-60% 1RM).
- Synergy 3 (posterior muscles): Eccentric-focused training targeting deceleration capabilities.

Program implementation should follow a 3:2:2 ratio of training volume between synergies 1, 2, and 3 during initial phases, shifting toward 2:3:3 as players advance, reflecting the greater importance of power generation and deceleration control at higher performance levels. This synergy-based approach fundamentally differs from traditional muscle-isolated training by emphasizing coordinated activation patterns rather than individual muscle development (43).

## 5 Conclusions

This investigation successfully quantified muscle synergies during the badminton forehand overhead smash through non-negative matrix factorization of EMG data and MSK modeling, providing novel insights into the neuromuscular control strategies underlying this complex, high-velocity movement. Three distinct synergies were identified: (1) trapezius-dominant synergy for scapular stabilization during acceleration, (2) pectoralis/anterior deltoid synergy for internal rotation and flexion during impact, and (3) posterior muscle synergy for controlled deceleration. The computational musculoskeletal model demonstrated strong agreement with experimental EMG data for most muscles, with notable exception of the lower trapezius. These findings highlight the integration of EMG and MSK modeling as a robust approach for analyzing neuromuscular control strategies in high-velocity overhead movements. Further research should expand to other overhead sports, explore alternative algorithms such as ICA or PCA, employ advanced EMG techniques for deep muscles, and conduct longitudinal studies examining how training or rehabilitation alters synergy structures, thereby enhancing applications in both athletic performance optimization and injury prevention. Future research should expand methodological approaches to include dynamic optimization algorithms that better account for force-velocity relationships and inertial effects during explosive movements. Such approaches may improve model predictions for muscles exhibiting complex activation patterns during rapid acceleration and deceleration phases.

## Data Availability

The datasets presented in this study can be found in online repositories. The names of the repository/repositories and accession number(s) can be found below: N/A.
